# Comparing microbiotas of foals and their mares’ milk in the first two weeks after birth

**DOI:** 10.1186/s12917-023-03864-1

**Published:** 2024-01-08

**Authors:** Michael J. Mienaltowski, Mitchell Callahan, Ubaldo De La Torre, Elizabeth A. Maga

**Affiliations:** 1 Department of Animal Science, University of California Davis, One Shields Avenue, 2251 Meyer Hall, Davis, CA 95616 USA; 2 Department of Medicine, Division of Pulmonary and Critical Care Medicine, Oregon Health & Science University, 3181 SW Sam Jackson Park Road, BRB 440, Portland, OR 97239 USA

**Keywords:** Mare’s milk, Lysozyme, Foal gut transitioning, Microbiota

## Abstract

**Background:**

The mare-foal relationship is essential for the well-being and growth of a foal. Mare’s milk provides a foal with nutrients, protective immunity, and microbes. Within the first two weeks of life, there is a risk for a foal to suffer from diarrhea, particularly “foal heat diarrhea” which happens at about the time of a mare’s estrus cycle but is more likely due to transitions in the microbiota in the foal’s gastrointestinal (GI) tract. We hypothesized that this GI microbiota transition could be caused by changes in lysozyme and microbial populations in the mare’s milk. To test this hypothesis, fifteen mare-foal pairs were followed in the first 15 days post-foaling. Every other day milk was collected from mares and rectal swabs were collected from foals. Lysozyme activity in the mare’s milk was measured using a fluorescence assay. Microbial DNA was isolated from the milk and swabs and the V4 domain of 16 S rRNA genes were PCR amplified and sequenced using Illumina MiSeq technology. Microbial populations were analyzed using *DADA2* and *phyloseq* within *R*.

**Results:**

Mare’s milk lysozyme activity peaked for samples at Day 1 and levels dropped to 72.5% of Day 1 activity by Day 15; however, microbial populations in the mare’s milk did not vary significantly over the two weeks. Furthermore, levels of microbial diversity found in foal rectal swabs were initially similar to microbial diversity seen in mare’s milk; however, over the first fifteen days, diversity increased for the foal rectal swab microbiota and swab microbial populations differed from milk microbes. A transition occurred shifting from microbes from the phylum *Proteobacteria* early in rectal swabs to those primarily from the phyla *Firmicutes* and *Bacteroidota* after the first few days post-foaling. These phyla contained several families and genera of microbes that promote utilization of milk components in healthy gut transition. Microbial abundance levels correlated more with days post-parturition than with lysozyme activity and mare’s milk microbial populations.

**Conclusions:**

The findings suggest that much of the microbial populations responsible for the transition of the foal’s gut comes from sources outside of mare’s milk species and levels of lysozyme activity.

**Supplementary Information:**

The online version contains supplementary material available at 10.1186/s12917-023-03864-1.

## Background

The nutritional status of the foal is crucial for its optimal development. Within the span of a 4–6 month window from foaling to weaning, a foal will grow to up to 50% of its body weight [1]. To meet requirements for such growth, a foal will transition from milk consumption to a forage and grain diet; thus, fecal microbial composition changes will occur where initial predominant populations have the ability to metabolize milk and then gut microbial populations change such that a weanling more efficiently utilizes fibrous plant material [2]. With the foal relying heavily upon mare’s milk, this food source is incredibly important to its growth. Both colostrum and milk provide energy, nutrients and non-nutritive components such as immunoglobulins, cells, enzymes, hormones, insulin-like growth factor 1 (IGF-1), and protective and trophic factors that play a role in immune competency, metabolism, musculoskeletal growth and disease prevention [3–6]. Mare’s milk also contains non-protein nitrogen, which is believed to play roles as taste factors and substrates for milk microbes [1, 3, 4, 7]. Many such similar factors have been well-studied in human breast milk, which contains diverse populations of bacteria that are hypothesized to seed the infant’s gut via breastfeeding [8]. It should be noted that microbial populations in expressed milk could come from within an enteromammary pathway, from the skin microbiome, as well as from environmental contamination [9]. That said, those factors in the breast milk seem to contribute to promoting gut health in the infant, especially early on allowing for microbes from the genera *Lactobacillus*, *Bacteroides*, and *Clostridia* to flourish [10]. Moreover, these factors in combination with the healthy gut microbes promote the activation of the infant’s immune system, improved intestinal epithelial tight junction barrier function, as well as improved mucous production, all of which contribute to healthy gut homeostasis in the neonate [10].

Besides nutrition for the foal and the microbes, mare’s milk provides a foal with other important naturally occurring biomolecules that contribute to immunity. Immunoglobulins and levels of innate antimicrobial molecules like lysozyme to help fend off some diarrheas [11]. When 8-week-old pigs consumed lysozyme-rich transgenic goat milk for two weeks, it was noted that levels of *Bacteroidetes* increased while levels *Firmicutes* (*Clostridia*) declined within that period of consuming the lysozyme-rich milk, relative to control goat milk [12]. Furthermore, in that time, pigs consuming lysozyme-rich transgenic goat milk had increased relative levels of microbes from *Bifidobacteriaceae* and *Lactobacillaceae* which are essential to pig gut health [12]. Lysozyme plays an important role in modulating the abundance of gut health-promoting microbes. Moreover, bacterial communities in the milk of equids, like other mammals, can provide the foal with suitable microbes for promotion of a healthy gut [8, 10]. It is essential that microbial richness be maintained in the gut of a healthy foal; foals with diarrhea have decreased levels of microbial diversity [13]. Diarrhea affects 60% of foals during their first six months of life [13]. Sometimes the diarrhea is caused by an infectious source, and often it occurs from self-limiting foal heat diarrhea in the first two weeks of life [13, 14]. Because diarrhea is so prevalent in foals early in life while suckling, changes in components of the mare’s milk early in the life of the foal could affect the foal gastrointestinal (GI) microbiota. We believe that changes in mare’s milk lysozyme levels could influence the foal’s GI microbiota.

In this study, we hypothesized that the mare’s milk microbiota affects the bacterial populations in the gut of healthy foals in the first fifteen days of life as a GI transition occurs. Moreover, we hypothesized that a change in lysozyme in the mare’s milk could affect gut microbiota of healthy foals in the first fifteen days of life. Lysozyme levels in mare’s milk were tracked over the first fifteen days post-foaling, and high-throughput next-generation sequencing (NGS) technology was used in order to determine how microbiome compositions compare over time between mare’s milk and foal rectal swabs.

## Results

### Analysis of lysozyme levels in mare’s milk

**Fig. 1:**
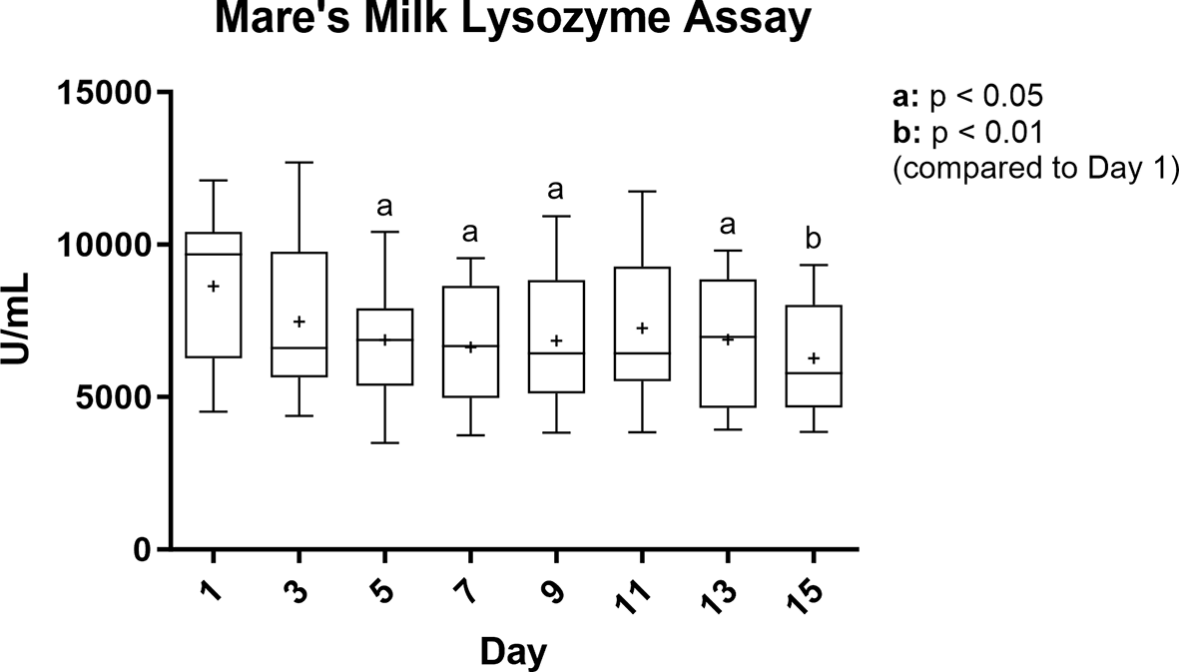
Analysis of lysozyme activity of mare’s milk reveals small but significant changes in lysozyme levels. Lysozyme activity levels were maximal in the first day after foaling; however, they steadily declined over 15 days. Data presented as a box and whisker plot with “+” representing mean; n = 14–15 mares in matched one-way ANOVA with Dunnett’s multiple test correction by day reported as adjusted p-value: a, p < 0.05 compared to Day 1; b, p < 0.01 compared to Day 1

Mares and foals in the study were either Quarter Horses or Thoroughbreds housed at one of two farms (Table S1). Lysozyme activity in mare’s milk was highest at Day 1 after foaling. Afterward, relative to Day 1, decreases in activity ranged from 86.5% of initial levels at Day 3 and 72.5% of initial levels at Day 15 (p < 0.01) (Fig. 1).

### Microbial diversity

**Fig. 2:**
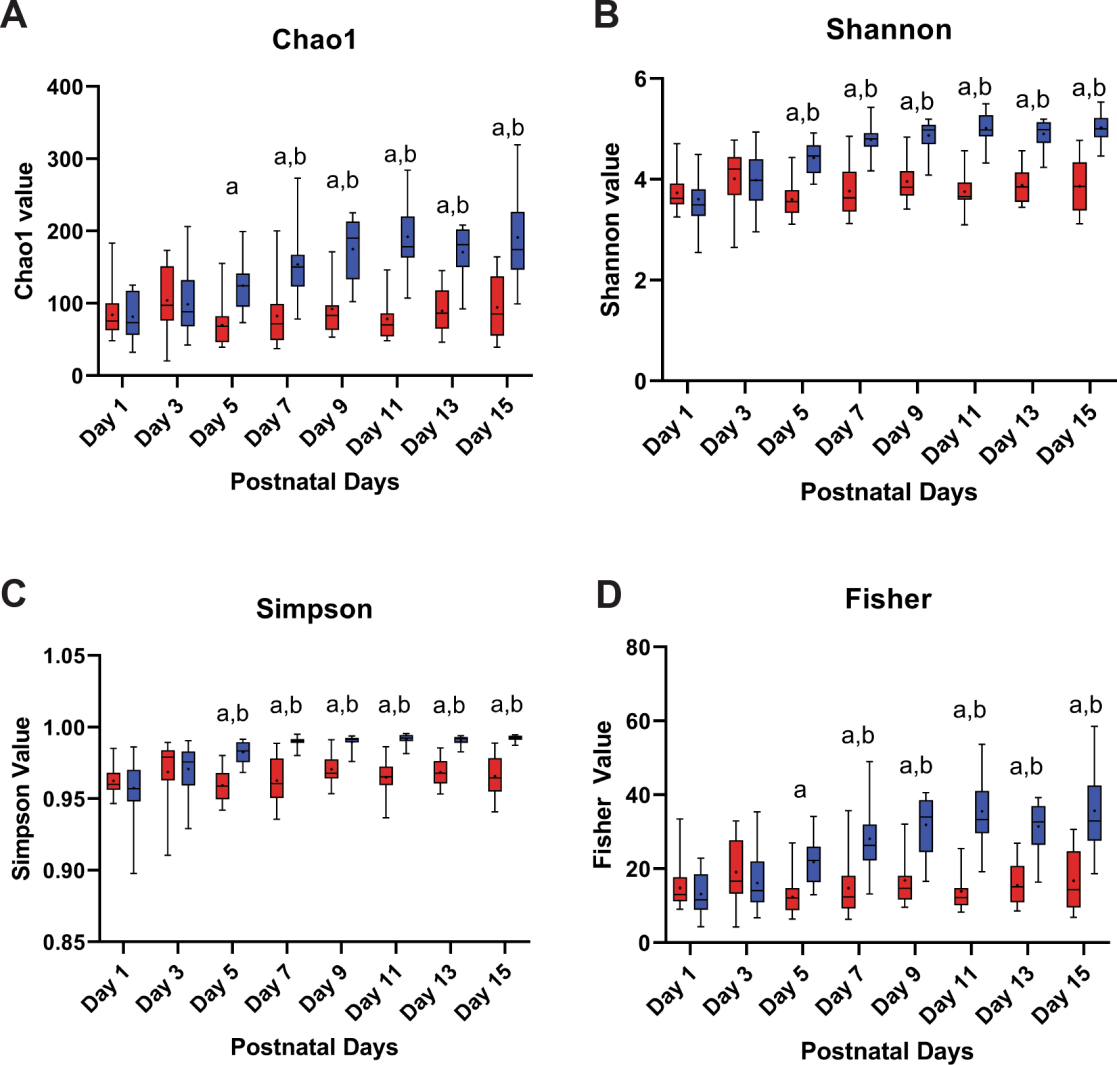
Diversity analyses of mare’s milk and foal rectal swabs reveal greater changes in diversity in swabs. Alpha diversity was analyzed by (**A**) Chao 1, (**B**) Shannon, (**C**) Simpson, and (**D**) Fisher indices. The red box plots represent mare’s milk, and the blue box plots represent foal rectal swab. By Day 5, all four diversity indices were greater for the foal rectal swab relative to mare’s milk. Amongst the foal rectal swabs, by Day 5 or 7, the diversity indices were significantly greater relative to those of rectal swabs within the first day of post-foaling. n = 13–15 mare’s milk and rectal swab sets; two-way ANOVA (Sample Type x Day) matched with repeated measures with post-hoc Tukey’s comparisons as p_adjusted_: a, milk vs. swab contrast p < 0.05. b, vs. Day 1 rectal swab p < 0.05

**Fig. 3:**
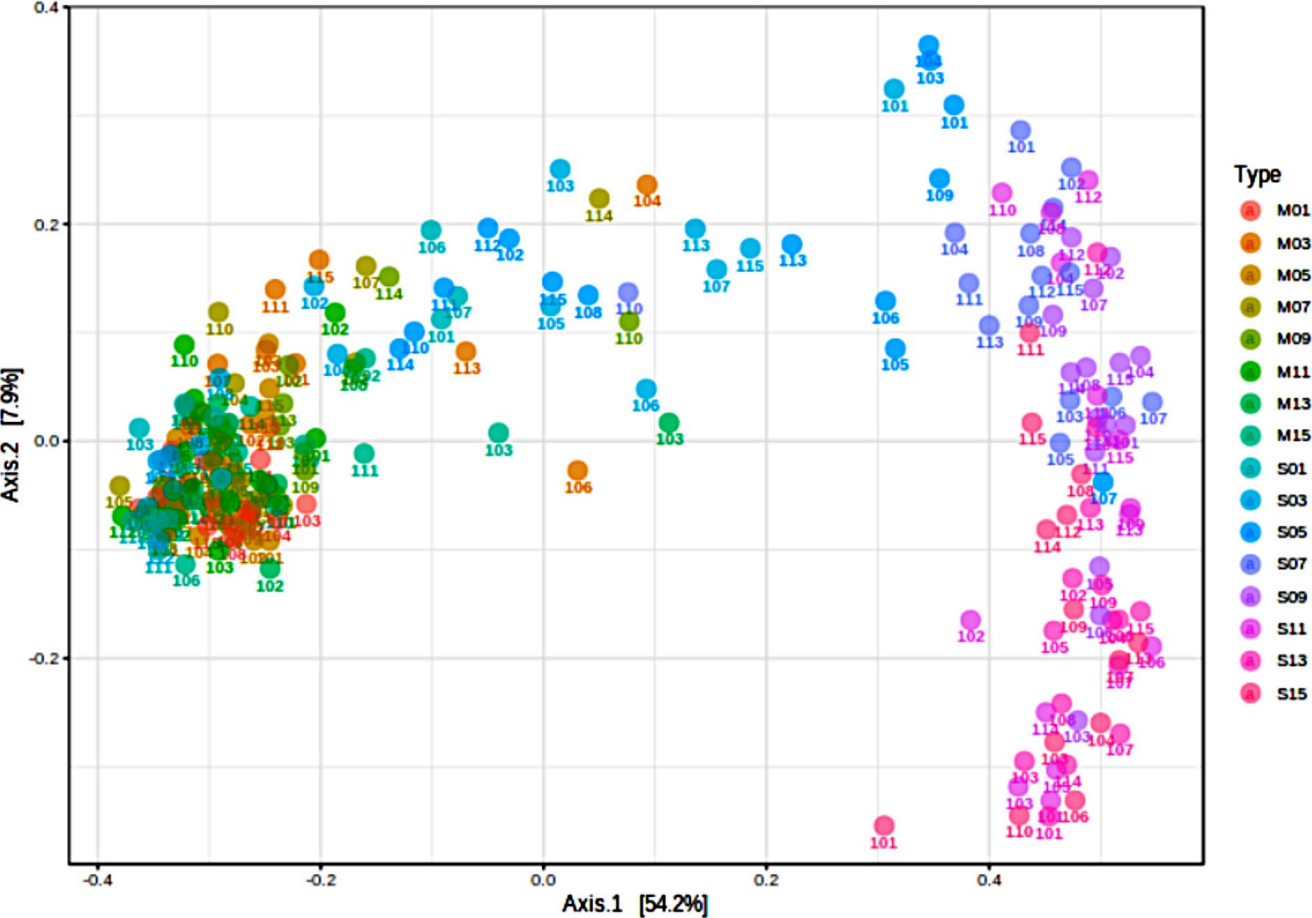
Principal coordinate analysis of microbial genera. Genus level data were applied for beta diversity profiling in a principal coordinate analysis plot using Bray-Curtis Index for the distance method, combined day and type as experimental factor, and PERMANOVA statistics. Each point represents a rectal swab or milk sample for each mare-foal pair, noted as Horse. Based on the key, type and day for each sample is noted (M, mare’s milk; S, foal rectal swab; days post-foaling are accompanying numbers); n = 234 samples presented (117 milk and 117 swab). The milk and swab samples cluster over the first five days, and then swab samples segregate from mare’s milk samples

Microbial diversity was analyzed from the sequencing reads of PCR amplified V4 domains of bacterial 16 S rRNA genes for the mare’s milk and foal rectal swab samples of fifteen mare and foal pairs. Alpha diversity was analyzed according to species richness with Chao1 index (Fig. 2A), richness and evenness with the Shannon index (Fig. 2B), diversity and abundance with the Simpson index (Fig. 2C), and numbers of species with the Fisher index (Fig. 2D). In the first three days of life in the foals, rectal swab microbial populations had similar levels of diversity when compared to mare’s milk. By Day 5, diversity was greater for the microbial populations found on the rectal swabs, relative to mare’s milk by both the number of species represented (Chao1) (p < 0.05) and relative to microbial populations swabbed in the rectum on Day 1 (Shannon) (p < 0.05) (Fig. 2A, B). Simpson’s and Fisher’s indices of alpha diversity also illustrated greater diversity of microbial populations with more even species proportions for rectal swabs relative to mare’s milk (p < 0.05) (Fig. 2C, D). Beta diversity depicted in a principal coordinate analysis plotted using Bray-Curtis Index demonstrated that over the first five days post-foaling more similarity was seen in microbial populations for the milk and rectal swab samples (Fig. 3). Most mare’s milk samples continued to cluster together along the primary axis of variation; by Day 7, foal’s rectal swabs segregated away from milk samples and demonstrated variation along Axis 2 (Fig. 3).

### Comparison of microbial populations amongst and between samples

**Fig. 4:**
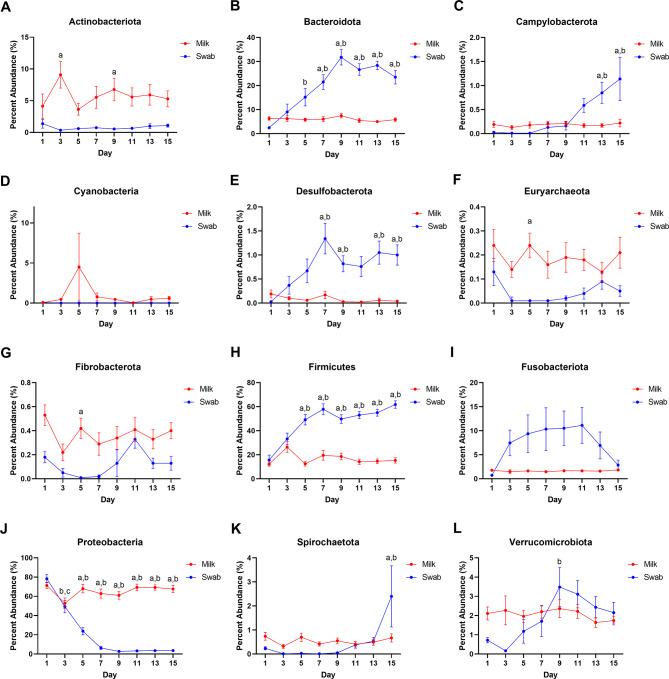
Distribution of microbial phyla. Changes in abundance were seen across the twelve predominant phyla: (**A**) *Actinobacteria*, (**B**) *Bacteroidota*, (**C**) *Campylobacterota*, (**D**) *Cyanobacteria*, (**E**) *Desulfobacterota*, (**F**) *Euryarcheota*, (**G**) *Fibrobacterota*, (**H**) *Firmicutes*, (**I**) *Fusobacteriota*, (**J**) *Protobacteria*, (**K**) *Spirochaetota*, and (**L**) *Verrucomicrobiota*. n = 13–15 mare’s milk and rectal swab sets; mixed-effects model (Sample Type x Day) matched with repeated measures with Tukey’s multiple comparison tests as p_adjusted_: a, milk vs. swab contrast p < 0.05; b, swab vs. Day 1 swab p < 0.05; c, milk vs. Day 1 milk p < 0.05

**Fig. 5:**
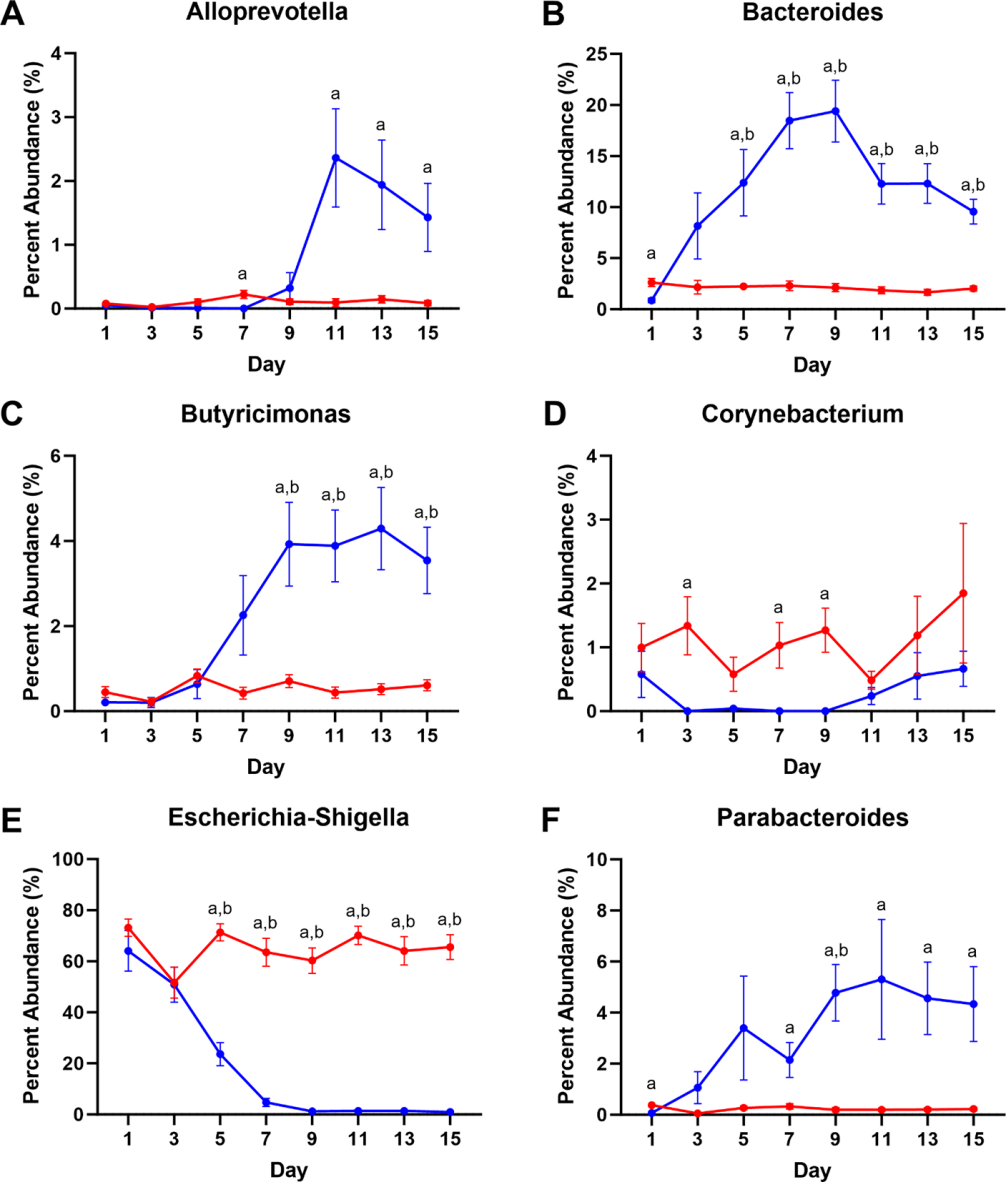
Distribution of microbial genera from Phyla Actinobacteriota, Bacteroidota, and Protobacteria. Changes in abundance were seen across genera: (**A**) *Alloprevotella*, (**B**) *Bacteroides*, (**C**) *Butyricimonas*, (**D**) *Corynebacterium*, (**E**) *Escherichia and Shigella*, and (**F**) *Parabacteroides*. n = 13–15 mare’s milk and rectal swab sets; mixed-effects model (Sample Type x Day) matched with repeated measures with Tukey’s multiple comparison tests as p_adjusted_: a, milk vs. swab contrast p < 0.05; b, swab vs. Day 1 swab p < 0.05; c, milk vs. Day 1 milk p < 0.05

From the sequencing, 451,011 total amplicon sequence variants (ASVs) were generated from the milk samples with a mean of 3855 ± 1605 ASVs per sample. For the rectal swabs, 717,016 total ASVs were generated with a mean of 6128 ± 2158 ASVs per sample. Differences in microbial abundance were examined for all phyla and families registering detected microbes, as well as the genera within those families with a mean abundance of 1% or more for all samples within either the swab or milk groups. Several phyla were found to have differences in abundance within sampling days and between sample types. For milk samples, microbial populations detected were primarily comprised of the phyla *Proteobacteria*, *Firmicutes*, *Bacteroidota*, and *Actinobacteriota*, with other minor phyla contributions (Fig. 4). Moreover, at various time points in the first fifteen days after foaling, the phyla *Proteobacteria*, *Actinobacteriota*, *Fibrobacterota*, and *Euryarcheota* were found with greater abundance in milk samples, relative to rectal swabs. For the phyla *Proteobacteria*, initially swabs and milk had the same levels; however, by Day 5 and thereafter, levels remained greater in the mare’s milk relative to foal rectal swabs (p < 0.05) (Fig. 4J). Bacteria from the family *Enterobacteriaceae* made the greatest contribution to phyla *Proteobacteria* particularly in milk throughout the fifteen days, with microbes from the genera *Escherchia* and *Shigella* representing as much as 95% of the microbes in any one milk sample (mean: 65%) (Fig. 5E). *Actinobacteriota* microbes were also found in greater abundance in milk samples throughout the first fifteen days with significance at post-natal days 3 and 9 (p < 0.05) (Fig. 4A). Families with greater abundance in the milk samples included *Micrococcaceae* at Days 3, 7, 9, and 11 (p < 0.05), as well as *Intrasporangiaceae* at Day 3 (p < 0.05); only *Corynebacterium* was detected at levels around 1–2% in mare’s milk at the genus level (Fig. 5D). Moreover, phyla *Euryarchaeota* and *Fibrobacteriota*, though greater in milk samples, were only minor contributors to overall mare’s milk microbiome with greater abundance noted for families *Methanobacteriaceae* and *Fibrobacteriaceae*, respectively, at Day 5, relative to milk samples (p < 0.05) (Fig. 4F, G, Table S2); no genera demonstrated abundance greater than 0.20% and 0.40%, respectively.

**Fig. 6:**
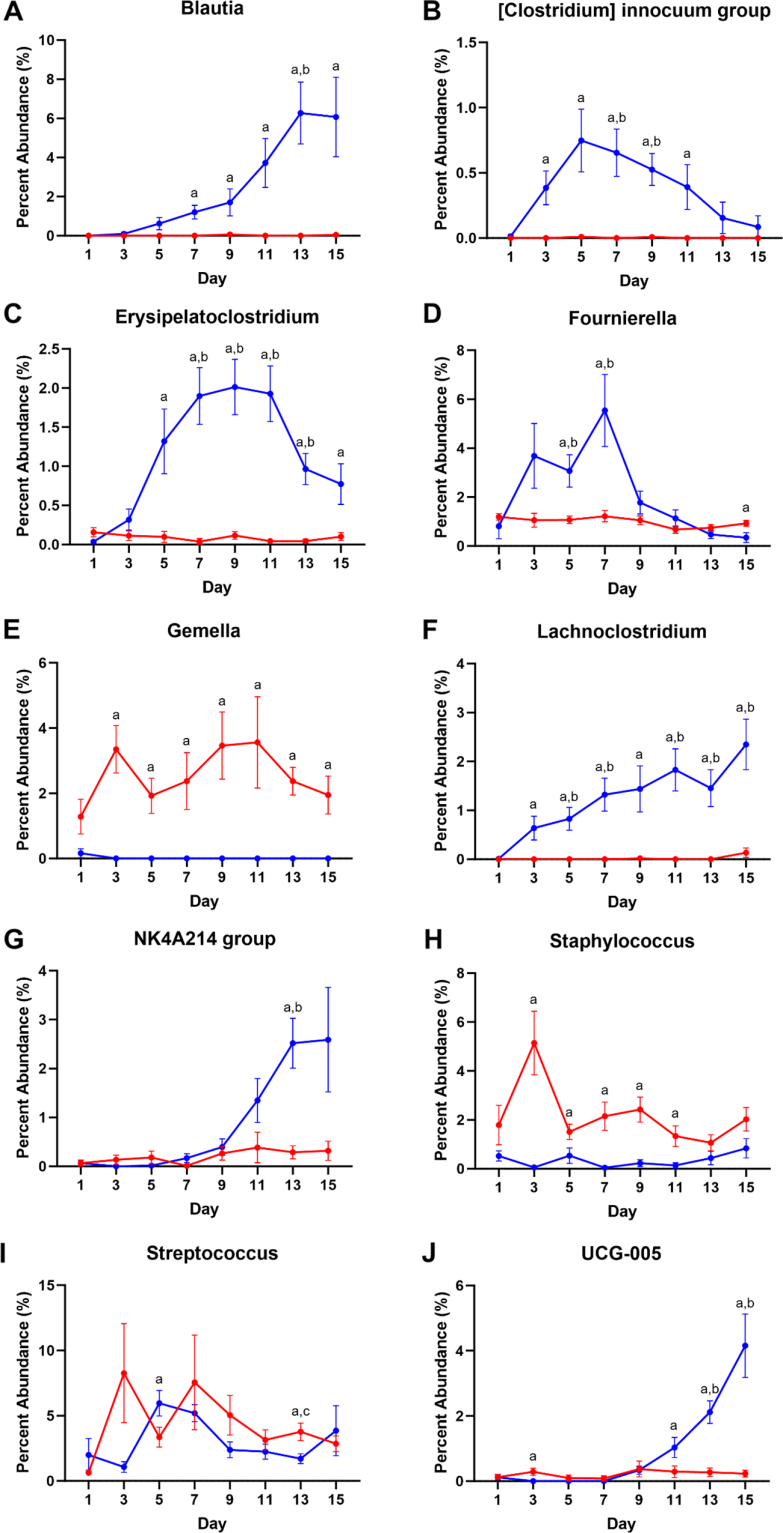
Distribution of microbial genera from phylum Firmicutes. Changes in abundance were seen across genera: (**A**) *Blautia*, (**B**) Clostridium innocuum group, (**C**) *Erysipelatoclostridium*, (**D**) *Fournierella*, (**E**) *Gemella*, (**F**) *Lachnoclostridium*, (**G**) NK4A214 group, (**H**) *Staphylococcus*, (**I**) *Streptococcus*, and (**J**) UCG-005. n = 13–15 mare’s milk and rectal swab sets; mixed-effects model (Sample Type x Day) matched with repeated measures with Tukey’s multiple comparison tests as p_adjusted_: a, milk vs. swab contrast p < 0.05; b, swab vs. Day 1 swab p < 0.05; c, milk vs. Day 1 milk p < 0.05

For rectal swab samples, there was a transition that occurred for microbial phyla from just after foaling to fifteen days post-foaling. In the first five days, the primary phylum detected in rectal swabs was *Proteobacteria*, particularly microbes from the genera *Escherchia* and *Shigella* (Fig. 5E); these levels decreased while levels from phyla *Bacteroidota* and *Firmicutes* steadily increased over fifteen days (Fig. 4B, H, J). For the rectal swabs, microbial abundance increased to levels greater than what was detected for milk for phyla *Bacteroidota*, *Campylobacterota*, *Desulfobacterota*, *Fimicutes*, *Spirochaetota*, and *Verrucomicrobiota*. For phylum *Bacteroidota*, abundance increased significantly by Day 5 (p < 0.05) (Fig. 4B) with significant contributions from microbes in the family *Bacteroidaceae* with families *Marinifilaceae*, *Prevotellaceae*, and *Tannerelaceae* starting at Days 9 or 11 (p < 0.05) (Table S2), particularly from genera *Bacteroides*, *Butyricimonas*, *Alloprevotella*, and *Parabacteroides*, respectively (p < 0.05) (Fig. 5). Likewise, for phylum *Firmicutes*, abundance increased from Day 1 reaching significance by Day 5 and then throughout the fifteen days (p < 0.05) (Fig. 4H); within the phylum, levels of microbes from the families *Lachnospiraceae* and *Oscillospiraceae* increased throughout the fifteen days. Genera contributing to this increase included *Blautia*, *Lachnoclostridium*, and the NK4A214 and UCG-005 groups of *Oscillospiraceae* (Fig. 6A, F, G, J). Additionally, within the phylum *Firmicutes*, families like *Eubacteriaceae*, *Ruminococcaceae* (genus *Fournierella*), *Erysipelatoclostridiaceae* (genus *Erysipelatoclostridium*), and *Erysipelotrichaceae* (Clostridium innocuum group) were generally more abundant in rectal swab samples (Fig. 6B–D). Moreover, levels of microbes from the genera *Staphylococcus* and *Gemella* were greater in mare’s milk (Fig. 6E, H); levels of microbes from the genus *Streptococcus* varied greatly from day to day between groups (Fig. 6I). Finally, increased abundance seen for phylum *Verrucomicrobiota* (Fig. 4L) came from genus *Akkermansia*, for phylum *Campylobacterota* came from genus *Campylobacter*, and for phylum *Desulfobacterota* was primarily genus *Desulfovibrio* (data not shown).

During the course of the fifteen days post-foaling, four foals presented with diarrhea: Horse 108 (Day 13), Horse 110 (Days 11, 13, 15), Horse 113 (Days11, 13, 15), and Horse 115 (Day 15). None of the foals with diarrhea were treated with antibiotics; however, a fifth horse – Horse 103 – presented with an umbilical infection from Days 9–15 and received trimethoprim-sulfamethoxazole during that period of time. An examination of a three-dimensional projection of the beta diversity PCoA plot (Fig. S1) demonstrated that the rectal swab samples from the horses with diarrhea at time of swabbing were still aligned with samples from healthy foals. The exception was the sample from Horse 115 at Day 15, which deviated from the other swab samples; the sample had a greater abundance of microbes from the genus *Streptococcus* than all other swabs at the time point (Horse 115: 22.78%; mean of other samples: 2.28%, with a range of 0–14.73%).

Samples were also compared between farms. The only phylum in which there was a difference was *Fusobacteriota* for rectal swabs. Rectal swabs from foals at Farm A had much higher levels of *Fusobacteriota* populations on Days 3, 5, and 7; however, after reaching maximum levels at Day 7 for Farm A, they decreased from Days 9–15. For Farm B, levels began to rise from Days 7 to 11, but then also decreased until Day 15 (Fig. S2). No statistically significant between-farm differences were found for genera *Cetobacterium* and *Fusobacterium* within the phylum, nor any other genera examined in the study (data not shown).

### Correlating genera of rectal swab microbial populations with lysozyme levels, mare’s milk microbial populations, and days post-foaling

Repeated measure correlations were determined for genera from microbial populations of the foal rectal swabs, relative to lysozyme activity levels, microbial populations of the mare’s milk, and days post-foaling (Table 1). Levels of *Escherichia* and *Shigella* were moderately positively correlated with lysozyme levels and very strongly negatively correlated with days post-foaling (p < 0.01).

**Table 1:** Repeated measures correlations

Family	Genus	Lysozyme r_rm_	Milk r_rm_	Day r_rm_
*Akkermansiaceae*	*Akkermansia*	−0.138	−0.054	**0.251** ^a^
*Bacteroidaceae*	*Bacteroides*	−0.190	−0.115	**0.226** ^a^
*Butyricicoccaceae*	*Butyricoccus*	−0.177	0.033	−0.105
*Campylobacteraceae*	*Campylobacter*	**−0.277** ^b^	0.005	**0.462** ^b^
*Christensenellaceae*	*Christensenellaceae R-7 group*	−0.086	0.281	**0.477** ^b^
*Corynebacteriaceae*	*Corynebacterium*	−0.105	0.086	0.124
*Desulfovibrionaceae*	*Bilophila*	−0.200	−0.036	−0.020
	*Desulfovibrio*	−0.151	−0.127	**0.408** ^b^
*Enterobacteriaceae*	*Escherichia-Shigella*	**0.569** ^b^	0.066	**−0.765** ^b^
*Enterococcaceae*	*Enterococcus*	0.129	0.008	**−0.277** ^b^
*Erysipelatoclostridiaceae*	*Erysipelatoclostridium*	−0.174	−0.052	**0.225** ^a^
*Erysipelotrichaceae*	*[Clostridium] innocuum group*	**−0.456** ^b^	−0.025	−0.051
*Fusobacteriaceae*	*Cetobacterium*	−0.067	−0.086	0.033
	*Fusobacterium*	**−0.335** ^b^	−0.110	−0.057
*Lachnospiraceae*	*[Ruminococcus] gnavus group*	−0.169	0.007	**−0.224** ^a^
	*[Ruminococcus] torques group*	−0.202	−0.044	0.099
	*Blautia*	−0.099	0.006	**0.561** ^b^
	*Lachnoclostridium*	**−0.271** ^b^	0.123	**0.487** ^b^
	*Marvinbryantia*	−0.074	**0.298** ^b^	**0.345** ^b^
*Lactobacillaceae*	*HT002*	**−0.238** ^a^	0.125	0.172
	*Lactobacillus*	**−0.211** ^a^	−0.06	**0.337** ^b^
	*Ligilactobacillus*	−0.168	−0.043	**0.345** ^b^
*Marinifilaceae*	*Butryicimonas*	−0.158	0.153	**0.540** ^b^
*Oscillospiraceae*	*Colidextribacter*	−0.055	−0.03	**0.403** ^b^
	*Flavonifractor*	−0.187	0.146	**−0.268** ^b^
	*NK4A214 group*	−0.033	0.094	**0.549** ^b^
	*UCG-005*	−0.124	−0.024	**0.613** ^b^
*Prevotellaceae*	*Alloprevotella*	−0.088	−0.003	**0.476** ^b^
	*Prevotella*	−0.081	0.066	**0.298** ^b^
	*Prevotella UCG-001*	−0.083	**0.199** ^a^	**0.314** ^b^
	*Prevotella UCG-003*	−0.007	−0.023	**0.283** ^b^
	*Prevotella UCG-004*	−0.027	−0.148	**0.272** ^b^
*Ruminococcaceae*	*Fournierella*	−0.029	**0.205** ^a^	**−0.295** ^b^
	*Ruminococcus*	−0.116	−0.001	**0.397** ^b^
*Staphylococcaceae*	*Staphylococcus*	−0.071	−0.024	0.063
*Streptococcaceae*	*Streptococcus*	−0.152	0.102	−0.001
*Sutterellaceae*	*Sutterella*	**−0.366** ^b^	0.068	0.125
*Tannerellaceae*	*Parabacteroides*	−0.088	0.074	**0.298** ^b^

Repeated measure correlation, r_rm_; p < 0.05, ^a^p < 0.01, ^b^significant correlations **bolded**

Levels of Clostridium innocuum group were moderately negatively correlated with lysozyme levels (p < 0.01). *Sutterella*, *Fusobacterium*, *Campylobacter*, *Lachnoclostridium*, and *Lactobacillaceae* family HT002 and *Lactobacillus* were weakly negatively correlated with lysozyme levels (p < 0.05). *Prevotella* UCG-001, *Fournierella*, and *Marvinbryantia* were weakly positively correlated with mare’s milk microbe levels (p < 0.05). Levels for many genera were weakly positively and negatively correlated with days post-foaling (Table 1). The strongest correlations with days post-foaling were *Escherichia* and *Shigella* (negative), *Oscillospiraceae* family UCG-005 (positive), *Blautia* (positive), *Oscillospiraceae* family NK4A214 group (positive), and *Butyricimonas* (positive) (p < 0.01).

## Discussion

Throughout the first fifteen days post-foaling, we found that there were changes in the levels of lysozyme in mare’s milk, yet there was not much change to the microbial populations detected in the milk, neither for diversity indices nor for specific microbial populations. A previous study following eight Quarter Horse mares at three time points (36 h, 70 days, and 136 days) also found no significant changes to microbial population levels in mare’s milk during lactation [15]. In the first two weeks of nursing, mare’s milk has been found to be rich in dry matter, protein, and gross energy; then throughout lactation, protein and fat content reduce while sugar content increases, though gross energy does decrease overall [16]. Thus, it seems that the changes in sugar, protein, and fat composition (~ 21%, ~ 18%, ~ 17%, respectively) do not have a dramatic effect on the microbial population within the milk [15, 16]. Factors like immunoglobulins, lactoferrin, and lysozyme are also important components in mare’s milk, particularly for the foal. We found a decrease in lysozyme over time such that by Day 15 lysozyme activity was only at 72.5% of Day 1’s levels. Like lysozyme, levels of lactoferrin have been found by others to be at their maximum early in lactation with decreases over time [17]. Similarly, for immunoglobulins, levels are greatest in colostrum from which the foal suckles in the first 24 h; levels then decrease in mare’s milk by half within the first month and then ever further over time to less than 30% of original levels at 5 months [18]. Factors like lysozyme, lactoferrin, and immunoglobulins have all been described as essential for maintenance of a healthy gut; thus, while the horse is suckling from the mare, these factors should promote foal growth, performance, and health [4, 17, 19, 20]. Correspondingly, these essential factors in the milk are then ingested by the foal and contribute to immunological status of the foal; thus, with levels of colostrum, lysozyme, and lactoferrin decreasing over time, such changes could impact microbial populations in the foal GI.

Unlike with mare’s milk, foal rectal swab microbial populations did change over the first fifteen days post-foaling. Over the first three days, levels of microbial diversity were similar for rectal swabs and mare’s milk. The PCoA demonstrated that microbial populations, even at the genus level were similar the first three days for both sample sets. Microbial populations of the rectal swabs and milk samples were rich in microbes from the phylum *Proteobacteria* over the first few days, with microbes from *Escherichia-Shigella* being most abundant in both sample sets. Microbes like these from the family *Enterobacteriaceae* are typically found in raw milk in many species like horses and cows, which is the reasoning behind the pasteurization process for safe human consumption [21–23]. Moreover, levels of *Escherichia-Shigella* from the rectal swabs were moderately positively correlated with milk lysozyme levels but very strongly negatively correlated with days post-foaling, which is suggestive that lysozyme might not play a major direct effect on changes seen in levels of *Escherichia-Shigella* or that lysozyme activity though decreasing is still effective at reducing microbes within the genus. Three other genera with levels that were weakly negatively correlated with lysozyme activity were *Sutterella*, *Fusobacterium*, and *Campylobacter*. Microbes from these three genera have been associated with inflammatory bowel disease, colitis, and hemorrhagic diarrhea; thus, lysozyme activity could play some role in mitigating levels within these three genera [24–26].

After the first few days post-foaling, there was a transition away from the phylum *Proteobacteria* to genera primarily from the phyla *Firmicutes* and *Bacteroidota*, which matches transitions seen in our previous study and those of others [2, 27]. By about Day 7, rectal swab microbes in the phylum *Firmicutes* increased from about 20% abundance to nearly 60%. Several families important for gut colonization and a healthy gut transition, like *Lachnospiraceae* and *Lactobacillaceae*, had increased abundance. Levels for genera like *Blautia* and *Lachnoclostridium* were moderately positively correlated with days post-parturition, while *Marvinbryantia*, *Ligilactobacillus*, and *Lactobacillus* were weakly positively correlated with days post-foaling. Microbes in these families increase in response to the complex carbohydrates in the foal’s diet, including forage nutrients like cellulose [2, 28, 29]. Moreover, levels of *Oscillospiraceae* – in particular *UCG-005*, *NK4A214*, and *Colidextribacter* – increased later in the first two weeks of the foals’ lives; microbes from the family *Oscillospiraceae* have been associated with normal feces in calves [30]. Furthermore, relative to other microbial families, levels of *Ruminococcus* were weakly positively correlated with days post-parturition. Within *Firmicutes*, rectal swab levels for microbes from genera *Fournierella* and *Marvinbryantia* were weakly positively correlated with level of those genera in mare’s milk. Studies have found these genera in milk from humans and rats [31, 32]. Thus, for the most part, changes within *Firmicutes* seemed to be better correlated with aging than with milk or lysozyme activity. In that two week period, microbial enrichment of *Firmicutes* is more likely coming from access to the mare’s feed and feces [2, 28].

We found that no microbial family within the phylum *Bacteroidota* comprised more than roughly 2% of mare’s milk microbiome, though *Bacteroidetes* levels increased in foal rectal samples over time. Similar low abundance of *Bacteroidetes* has also been found in human milk [33]. *Bacteroidetes* have been found in the mare’s vagina and to a lesser extent in her oral cavity, though they are also commonly found in the soil [27, 34]. In the foal rectal swabs, levels of *Bacteroidetes* increased over the first nine days post-foaling to 30% abundance. In human infants, *Bacteroides* species have been found to flourish in the GI due to milk oligosaccharides; likewise, in the horse, this interaction ultimately promotes a healthy gut and future solid food consumption like the mare’s food, including hay and grain [35, 36]. Levels of genus *Bacteroides* increased to as much as 20% abundance and were weakly positively correlated with days post-parturition, but not at all correlated with milk *Bacteroides* levels or lysozyme activity. Levels of *Alloprevotella* were moderately positively associated with days post-foaling; levels of *Prevotella* and its several groups were weakly associated with days post-foaling. Microbes from the *Prevotellaceae* family have been found in animals with a concentrate-eating enterotype rich in carbohydrates, which could be indicative of initiation of eating solid foods [37, 38]. Within the *Prevotellaceae* family, only *Prevotella UCG-001* was very weakly correlated with levels of the same genus in milk. Within the family *Marinifilaceae*, levels from the genus *Butryicimonas* were moderately positively correlated with days post-parturition. Abundance of this family has been shown to rise during suckling in other species, peaking at a time when gut barrier formation is signaled to initiate from microbial metabolites, and then falling with solid food ingestion [39]. Thus, while milk is likely not the source of *Bacteroidetes*, within milk are oligosaccharides that provide essential resources for those microbes to cultivate as foals transition to solid food ingestion.

Interesting findings were also seen in the phyla *Verrucomicrobiota*. Within phylum *Verrucomicrobiota*, abundance levels in foal rectal swabs increased over time until eclipsing milk samples before then falling to the levels in mare’s milk. This was also seen at the family level (*Akkermansiaceae*) and genus level (*Akkermansia – data not shown*). Microbes from *Verrucomicrobiota* have been found in human and donkey milk with extracellular vesicles containing these microbes detected in the milk of healthy mothers, thus suggesting a source for postnatal gut colonization [40, 41]. Within the phylum, species from the family *Akkermansiaceae* have been found in the rumen fluid of neonatal calves despite being at a stage in which the rumen is inactive [42]. Moreover, species within this family have been found within the GI of human infants and foals, likely because of the increased growth of *Akkermansiaceae* species utilizing milk oligosaccharides as a nutritional source [35, 43].

This study had several limitations. Mare’s milk nutrient composition was not analyzed; instead, we focused on levels of lysozyme and microbes in expressed mare’s milk. However, it would be beneficial to analyze the nutrient composition in mare’s milk in our future studies considering this early timeframe post-foaling. There would be value in correlating nutrient composition in milk with shifts in microbial populations. Moreover, it would be useful to analyze nutrient composition of the forage and feeds to which the foals were exposed, as well as discern which microbes found in soil on farm could also be a seed source. Likewise, using labeled bacteria to track colonization of the foal gut would also be valuable. Furthermore, we amplified and sequenced the V4 domain of bacterial 16 S rRNA genes to identify microbial populations present in milk and rectal swabs. While this is certainly of value to understanding the microbial populations present, there would be more power to analyzing the microbial transcriptome collectively expressed by microbes in the mare’s milk, foal rectal swabs, even the contaminants from mare’s udders and soil to understand active processes ongoing during nursing of a newborn foal and to determine if the microbes are alive, intact, and or active. We will take these limitations into consideration in planning follow-up studies.

## Conclusions

From this study, we were able to draw several conclusions. Firstly, over the first fifteen days post-foaling, the microbial populations in the mare’s milk do not change significantly, yet a significant difference was noted in the lysozyme activity detected. Secondly, the levels of diversity of microbial species found in the foal’s rectal swab initially was similar to that of mare’s milk. However, over the first fifteen days, diversity increased for the foal rectal swab microbiota. Transitions were seen in the foals’ GI microbiota over time, and the transition of a few microbes could be correlated to milk microbes or lysozyme levels; however, more and stronger correlations were seen when considering days post-parturition. This is highly suggestive that microbes likely derive from other sources like solids whether food, feces, and/or soil. Over the first few days post-foaling, microbes from the phylum *Proteobacteria* were predominant, especially the genus *Escherichia-Shigella*, which was also the predominant genus in the expressed milk. However, after the first few days, a transition occurred to bacteria primarily from the phyla *Firmicutes* and *Bacteroidota*, which contain several genera of microbes that promote utilization of milk components and the transition to gut microbes necessary for solid food like forage and creep feed. From this study, we can conjecture that the mare’s milk microbes and lysozyme levels play a role, albeit small based upon our findings. Future studies should focus on the role of milk nutrient composition on the transitioning gut of the foal as well as microbes found in other sources to which the foal is exposed.

## Materials and methods

### Horses and sample collection

Research was conducted with the approval of the UC Davis Institutional Animal Care and Use Committee. Mares and foals were either Quarter Horses or Thoroughbreds housed at one of two farms; seven sets of mares and foals were from Farm A and eight sets of mares and foals were from Farm B (Table S1). Management for the farms had similarities and differences; mares were fed combinations of hay and supplemental grain; at the second farm, the horses had daily access to pasture (Table 2) to provide nutrient requirements as recommended by the Committee on Nutrient Requirements of Horses for mares in their first month of lactation [44, 45]. Rectal swabs (FLOQSwabs 552 C, Copan Diagnostics Inc.) were taken from foals and mare’s milk was collected from their mares, every two days in the first fifteen days after foaling (days 1, 3, 5, 7, 9, 11, 13, 15) at both farms. A total of 117 milk samples and 117 rectal swabs were collected (Table S3). Samples were collected between 7:00 a.m. and 3:00 p.m. local time on collection days. Milk was manually expressed from mares while wearing examination gloves. Rectal swabs and milk samples were stored at −80 °C.

**Table 2:** Farm management

Farm	Housing/pastures	Nutrition
A	Days 0–7: Housed in stalls Days 7–15: Housed in stalls and/or dirt pens depending upon weather	Commercial pelleted feed (12–14% protein, 6–16% fiber with vitamins and minerals); No more than 50% alfalfa hay with teff hay as the remainder. Amount fed based upon body condition score of lactating mare
B	Days 0–3: Housed in stalls Days 3–15: Housed in stalls overnight. Stall fed in the mornings and evenings with pasture management between stall feedings	In stall twice daily: 3.8 L volume of barley, corn, electrolytes, vegetable oil, flaxseed; 5 kg alfalfa hay. In pasture: abundant pasture, elevated feeders with free choice alfalfa

### Lysozyme assay

Lysozyme activity in the mare’s milk was measured using the EnzChek® Lysozyme Assay Kit (Molecular Probes, #E-22,013) according to the manufacturer’s instructions. Briefly, activity from mare’s milk was compared to a chicken egg white control lysozyme activity standard curve [46]. The substrate on which lysozyme activity was tested was fluorescein-conjugated *Micrococcus lysodeikticus*. Digestion products were measured by absorption maxima at 494 nm and fluorescence emission maxima at 518 nm using a Synergy HT plate reader (BioTek). After optimization, mare’s milk samples were diluted 29.8x and then assayed with three technical replicates. Means of technical replicates were used for each biological replicate.

### Bacterial DNA isolation and library preparation, and sequencing

DNA was isolated from rectal swabs using the Quick-DNA Fecal/Soil Microbe Miniprep Kit (Zymo Research, #D6010) and from milk samples using the Milk Bacterial DNA Isolation Kit (Norgen BioTek, #21,550). Briefly, rectal swabs were immersed in phosphate-buffered saline (PBS) to disperse fecal material from the swab into the PBS; then swabs were pressed against the sides of the microcentrifuge tubes to express residual PBS from the swabs [46]. The suspension was then vortexed and added to ZR BashingBead™ Lysis Tubes and samples were processed according to manufacturer’s guidelines with adaptations to increase centrifuge speeds [2, 47]. To isolate DNA from mare’s milk, samples were processed according to manufacturer’s instructions (Norgen BioTek). DNA concentrations were determined using a NanoDrop UV spectrophotometer (ThermoFisher Scientific) and the V4 domain of bacterial 16 S rRNA genes was amplified to generate libraries bar-coded by sample. Primers F515 (forward: 5′-GTGCCAGCMGCCGCGGTAA-3′) and R806 (5′-GGACTACHVGGGTWTCTAAT-3′) were used to amplify the V4 domain and included a unique 8 bp barcode on each forward primer [46, 48]. PCR was performed in triplicate in 25-ul reactions using GoTaq 2X Green Master Mix (Promega) and programmed to follow an initial step at 94 °C for 3 min, followed by 35 cycles of 94 °C for 45 s, 50 °C for 1 min, and 72 °C for 90 s, ending with a final extension at 72 °C for 10 min. PCR amplification success was examined via agarose gel electrophoresis after triplicate reactions were combined for each sample. No amplification was ever noted in the negative (no DNA) controls. Then PCR products were combined by equal volume and purified using QIAGEN’s PCR Purification Kit. Combined barcoded libraries were submitted to the University of California Davis Genome Center DNA Technologies Core for 250 bp paired-end sequencing using the Illumina MiSeq platform. Raw sequence data are freely available at the Sequence Read Archive (SRA): Bio Projects PRJNA899677 and PRJNA899997.

### Sequencing analysis

Sequencing files were demultiplexed using *Barcode Splitter* [49] in Galaxy [50]. Demultiplexed FASTQ files were migrated to an XSEDE (Extreme Science and Engineering Discovery Environment) JetStream cloud computer platform to work with BioConductor v3.12 and DADA2 v1.18 in RStudio 1.4, as well as to personal computers to also run Rstudio using R.4.1.0 [51, 52]. Within BioConductor, reads were applied to the DADA2 pipeline [53–55]. In DADA2, quality profiles for the reads were visualized with the *plotQualityProfile* command, and then the reads were filtered and trimmed (240 bp forward read, 160 bp reverse read) with *filterAndTrim* using standard filtering parameters. Within DADA2, machine-learning was used to learn error rates ahead of core sample inference with the *dada* command [53]. Denoised sequences were obtained by merging paired reads with *mergePairs*, and then an amplicon sequence variant table (ASV) was constructed and chimeric ASVs were removed. Taxonomies were assigned using the Silva 138.1 species assignment, and then data tables were imported into the phyloseq R package 1.38 [56] for use with Biostrings [57]. These packages were used to produce data tables with taxa annotations and discern alpha diversity. Genera level data tables were applied to the Microbiome Analyst tool’s Marker Data Profiling (minimum count: 4; low count filter: prevalence 20%; low variance filter: 10%, inter-quartile range; data normalization with rarefaction and total sum scaling) to determine beta diversity and display as Principal Coordinates Analysis using Bray-Curtis Index at the genus level [58–60].

### Statistical analysis

All results were analyzed using jamovi 1.8 and displayed using GraphPad Prism v.9.4. Lysozyme assay results were statistically analyzed by one-way ANOVA matched with repeated measures with a multiple test correction by day. Microbial diversity, richness, and abundance of microbial populations for mare’s milk and rectal swab sets were analyzed by two-way ANOVA or mixed-effects model (Sample Type x Day) matched with repeated measures with Tukey’s multiple comparison tests. Repeated measure correlation analyses of microbial populations, lysozyme activity, and days post-foaling were performed using online tool rmcorrShiny [61, 62].

### Electronic supplementary material

Below is the link to the electronic supplementary material.

**Supplementary Material 1:** Comparing microbiotas of foals and their mares’ milk in the first two weeks after birth

## Data Availability

Raw sequence data are freely available at the Sequence Read Archive (SRA): Bio Projects PRJNA899677 and PRJNA899997.

## Abbreviations

ANOVA: analysis of variance
ASV: amplicon sequence variant
DNA: deoxyribonucleic acid
GI: gastrointestinal
PBS: phosphate-buffered saline
PCoA: principal coordinate analysis
rRNA: ribosomal RNA
XSEDE: Extreme Science and Engineering Discovery Environment
