# Supplementary material for: Comparing microbiotas of foals and their mares’ milk in the first two weeks after birth
Source: BMC Vet Res. 2024 Jan 8;20:17. doi: 10.1186/s12917-023-03864-1 (PMC10775675; doi:10.1186/s12917-023-03864-1)
Supplement: Supplementary file 1 — Supplementary Material 1: Comparing microbiotas of foals and their mares’ milk in the first two weeks after birth [file 12917_2023_3864_MOESM1_ESM.pdf]

Supplemental Materials

Table S1. Breed and Farm Information

**Horse Pair ID**	**Breed**	**Farm**
101	TB	A
102	QH	A
103	QH	A
104	TB	A
105	QH	A
106	QH Paint	A
107	QH	A
108	TB	B
109	TB	B
110	TB	B
111	TB	B
112	TB	B
113	TB	B
114	TB	B
115	TB	B

TB, Thoroughbred; QH, Quarter Horse

Table S2. Percentage abundance of microbial families present in mare's milk and foal rectal swabs.

Families		Day 1	Day 3	Day 5	Day 7	Day 9	Day 11	Day 13	Day 15
Phylum Firmicutes									
Acidaminococcaceae	Milk	0.03±0.10	0.06±0.13	0.12±0.27	0.07±0.18	0.05±0.20	0.07±0.17	0.02±0.09	0.09±0.18
	Swab	0.01±0.03	0.03±0.06	0.11±0.16	0.26±0.26	0.43±0.49	0.50±0.29	0.51±0.34	0.47±0.40
	Stats					ab	ab	ab	ab
Anaerovoracaceae	Milk	0.07±0.11	0.18±0.65	0.05±0.14	0.04±0.11	0.25±0.83	0.11±0.30	0.12±0.29	0.14±0.25
	Swab	0.07±0.19	0±0	0.02±0.09	0.56±0.62	0.30±0.50	0.48±0.62	0.98±0.90	1.13±0.99
	Stats				a			ab	ab
Butyricicoccaceae	Milk	0.82±0.61	0.49±0.47	0.61±0.53	0.71±0.58	0.68±0.43	0.42±0.56	0.57±0.44	0.77±0.42
	Swab	0.41±0.80	4.53±9.17	4.21±4.93	4.51±4.48	2.68±1.46	2.41±0.81	1.95±0.97	1.62±0.66
	Stats		ab	b	ab				
Christensenellaceae	Milk	0.19±0.42	0.14±0.26	0.25±0.42	0.16±0.29	0.09±0.16	0.01±0.03	0.18±0.37	0.12±0.25
	Swab	0.06±0.14	0.03±0.10	0±0.01	0±0	0.0±0.01	0.53±0.95	1.33±1.74	2.91±4.10
	Stats								ab
Clostridiaceae	Milk	0.03±0.10	0.14±0.23	0.04±0.17	0.16±0.30	0.22±0.33	0±0	0.16±0.37	0.22±0.32
	Swab	4.44±9.74	1.93±1.82	0.55±0.81	0.48±0.67	0.35±0.81	0.22±0.48	0.37±0.55	0.79±1.09
	Stats	a	b	b	b	b	b	b	b
Erysipelatoclostridiaceae	Milk	0.20±0.19	0.11±0.21	0.09±0.24	0.10±0.18	0.24±0.26	0.10±0.13	0.10±0.13	0.16±0.20
	Swab	0.06±0.11	0.28±0.45	1.28±1.49	1.84±1.21	1.72±0.90	1.94±0.92	1.64±0.81	1.82±0.93
	Stats		b	ab	ab	b	ab	b	b
Erysipelotrichaceae	Milk	0.11±0.13	0.01±0.04	0.05±0.10	0.04±0.12	0.06±0.11	0.11±0.11	0.05±0.09	0.07±0.09
	Swab	0.09±0.22	0.40±0.45	0.68±0.83	0.67±0.70	0.54±0.48	0.77±0.73	0.51±0.52	0.33±0.45
	Stats			ab	ab	a	ab		
Eubacteriaceae	Milk	0.12±0.31	0.08±0.18	0.07±0.18	0.06±0.15	0.06±0.15	0.08±0.21	0.05±0.10	0.07±0.16
	Swab	0.05±0.15	0.38±0.77	0.91±1.32	1.38±1.21	1.22±0.96	1.05±0.73	0.92±1.11	1.08±0.96
	Stats				ab	ab	ab		ab
Family XI	Milk	0.07±0.13	0.12±0.33	0.03±0.07	0.03±0.11	0.03±0.11	0.01±0.03	0±0	0.06±0.14
	Swab	0±0.01	0.03±0.13	0.08±0.32	0.11±0.42	0.16±0.53	0.21±0.45	0.22±0.43	0.48±0.95
	Stats								b
Gemellaceae	Milk	1.20±1.87	2.88±2.45	1.77±1.95	2.14±2.90	3.20±3.75	3.29±5.01	2.23±1.49	1.81±2.06
	Swab	0.15±0.49	0±0	0±0	0±0	0±0	0±0	0±0	0±0
	Stats								
Lachnospiraceae	Milk	1.66±1.05	1.83±2.38	1.34±1.10	1.01±1.10	1.69±1.14	1.28±1.51	1.13±0.91	1.74±1.22
	Swab	1.06±1.57	9.14±8.93	14.29±6.01	18.90±6.33	21.32±9.83	25.06±9.44	26.89±10.76	27.63±10.19
	Stats		b	ab	ab	ab	ab	ab	ab

Values presented as mean ± standard deviation. n = 13-15; a, $p < 0.05$ for milk vs. swab; b, $p < 0.05$ for swab vs. day 1; c, $p < 0.05$ for milk vs. day 1.

Table S2 (cont.). Percentage abundance of microbial families present in mare's milk and foal rectal swabs.

Families		Day 1	Day 3	Day 5	Day 7	Day 9	Day 11	Day 13	Day 15
Phylum Firmicutes (cont.)									
Lactobacillaceae	Milk	1.09±0.75	0.90±1.08	1.04±0.76	0.79±0.53	1.05±0.73	0.86±0.83	0.98±1.01	1.65±2.66
	Swab	0.93±1.64	0.62±2.10	3.86±4.34	7.53±4.86	7.77±4.26	4.55±2.26	4.21±2.43	4.43±3.73
	Stats				ab	ab	ab	a	b
Oscillospiraceae	Milk	0.56±0.82	0.63±0.98	0.51±0.69	0.40±0.60	0.90±1.72	0.84±1.68	0.86±0.99	0.72±0.83
	Swab	0.24±0.37	1.26±1.40	2.40±1.75	3.32±1.50	2.91±2.09	5.34±4.21	7.89±5.44	9.67±7.43
	Stats						ab	ab	ab
Peptococcaceae	Milk	0±0	0±0	0±0	0±0	0±0	0±0	0±0	0±0
	Swab	0±0	0.06±0.15	0.14±0.44	0.30±0.63	0.26±0.47	0.46±0.40	0.40±0.28	0.37±0.31
	Stats						ab	ab	ab
Peptostreptococcaceae	Milk	0.41±0.39	0.04±0.15	0.23±0.36	0.46±0.79	0.02±0.08	0.03±0.10	0.20±0.30	0.19±0.38
	Swab	3.30±6.79	3.87±2.52	3.13±2.07	1.67±0.91	1.59±0.92	1.41±0.76	0.72±0.75	0.29±0.58
	Stats	a	a	a				b	b
Planococcaceae	Milk	0.11±0.33	1.66±3.97	0.11±0.29	0.10±0.26	0.05±0.19	0.58±1.00	0.54±1.20	0.22±0.53
	Swab	0.33±0.86	0±0	0±0	0±0	0±0	0.06±0.22	0±0	0±0
	Stats		ac						
Ruminococcaceae	Milk	1.22±0.54	3.35±8.54	1.23±0.85	1.18±0.81	1.21±0.69	1.17±1.04	0.88±0.63	1.04±0.43
	Swab	0.97±2.52	4.39±5.56	7.23±5.40	10.66±8.16	5.36±3.93	5.10±3.89	3.82±2.40	3.40±4.70
	Stats			ab	ab				
Staphylococcaceae	Milk	3.13±4.87	6.16±4.92	2.08±1.25	3.36±3.95	3.54±3.04	1.92±2.35	1.77±2.21	2.67±2.40
	Swab	1.02±1.93	0.08±0.17	0.49±1.12	0.04±0.15	0.21±0.49	0.13±0.38	0.44±0.91	0.80±1.26
	Stats		a		a	a			
Streptococcaceae	Milk	0.57±0.76	7.25±12.90	3.05±2.65	6.91±12.56	4.51±4.94	2.87±2.70	3.54±2.36	2.66±2.17
	Swab	1.17±1.41	0.78±1.27	5.59±3.56	4.39±1.95	2.07±2.13	1.95±2.01	1.45±1.06	3.59±6.30
	Stats				b				
Phylum Verrucomicrobiota									
Akkermansiaceae	Milk	0.68±0.31	0.55±0.56	0.75±0.32	0.71±0.38	0.76±0.31	0.64±0.30	0.72±0.43	0.68±0.30
	Swab	0.30±0.32	0.17±0.26	1.08±2.38	1.61±3.16	3.49±4.00	2.73±2.78	1.86±1.99	1.19±1.94
	Stats					ab	b		
Phylum Bacteroidota									
Bacteroidaceae	Milk	2.45±1.40	1.84±1.96	2.07±0.96	2.12±1.61	1.98±1.41	1.72±1.20	1.57±1.05	1.90±0.89
	Swab	0.82±0.85	7.50±11.25	11.35±11.85	15.88±9.48	16.82±11.21	10.04±6.25	10.03±6.81	7.89±3.67
	Stats		b	ab	ab	ab	ab	ab	

Values presented as mean ± standard deviation. n = 13-15; a, $p < 0.05$ for milk vs. swab; b, $p < 0.05$ for swab vs. day 1; c, $p < 0.05$ for milk vs. day 1.

Table S2 (cont.). Percentage abundance of microbial families present in mare's milk and foal rectal swabs.

Families		Day 1	Day 3	Day 5	Day 7	Day 9	Day 11	Day 13	Day 15
Bacteroidota (cont.)									
Bacteroidales BS11 gut group	Milk	0.35±0.30	0.24±0.20	0.45±0.32	0.35±0.40	0.39±0.38	0.40±0.43	0.34±0.30	0.41±0.29
	Swab	0.07±0.09	0.03±0.07	0±0	0.01±0.02	0±0.02	0.24±0.62	0.20±0.39	0.37±0.70
	Stats			a					
Bacteroidales RF16 group	Milk	0.03±0.11	0.52±1.62	0.11±0.17	0.11±0.24	0.32±0.51	0.23±0.45	0.16±0.27	0.07±0.18
	Swab	0.05±0.11	0±0	0±0.008	0±0.015	0.14±0.56	0.26±0.45	0.27±0.42	0.30±0.57
	Stats								
F082	Milk	0.22±0.27	0.55±1.32	0.20±0.21	0.21±0.28	0.69±1.30	0.19±0.39	0.24±0.16	0.29±0.31
	Swab	0.11±0.16	0.01±0.02	0±0.015	0.±0.014	0.01±0.023	0.12±0.32	0.71±2.17	0.70±1.64
	Stats								
Marinifilaceae	Milk	0.44±0.45	0.21±0.33	0.78±0.60	0.42±0.52	0.72±0.60	0.42±0.44	0.52±0.46	0.62±0.42
	Swab	0.21±0.23	0.21±0.41	0.56±1.15	2.02±3.23	3.58±2.98	3.47±2.69	4.01±3.21	3.5±2.31
	Stats					ac	ac	ac	ac
p-251-o5	Milk	0.60±0.46	0.84±1.90	0.56±0.52	0.54±0.59	0.88±0.79	0.62±0.96	0.42±0.36	0.62±0.40
	Swab	0.32±0.33	0.08±0.14	0.02±0.05	0.02±0.05	0.25±0.90	1.20±1.63	2.62±4.43	0.43±0.55
	Stats							ac	
Prevotellaceae	Milk	0.42±0.30	0.50±0.74	0.47±0.37	0.42±0.39	0.41±0.39	0.46±0.56	0.44±0.28	0.38±0.31
	Swab	0.16±0.17	0.04±0.06	0.02±0.03	0.04±1.58	1.45±1.97	3.98±2.87	3.13±2.86	3.62±6.82
	Stats			a			ac	ac	ac
Rikenellaceae	Milk	0.72±0.46	0.68±0.93	0.48±0.52	0.94±0.70	1.18±1.17	0.64±0.71	0.66±0.55	0.81±0.93
	Swab	0.25±0.27	0.08±0.12	0.20±0.30	0.69±0.82	1.47±2.83	1.15±0.83	1.17±0.95	2.84±4.52
	Stats								ac
Tannerelaceae	Milk	0.70±0.42	0.30±0.36	0.48±0.34	0.53±0.36	0.49±0.32	0.33±0.25	0.43±0.27	0.35±0.21
	Swab	0.22±0.30	1.32±2.35	3.25±6.74	2.37±2.28	7.42±8.52	5.71±7.38	5.94±5.85	4.46±3.57
	Stats					ac	ac	ac	c
Phylum Proteobacteria									
Campylobacteraceae	Milk	0.19±0.23	0.13±0.17	0.14±0.19	0.20±0.23	0.22±0.21	0.17±0.20	0.17±0.15	0.14±0.20
	Swab	0.03±0.05	0.02±0.04	0.01±0.02	0.13±0.41	0.16±0.34	0.60±0.58	0.85±0.92	1.20±2.07
	Stats						c	c	c
Desulfovibrionaceae	Milk	0.17±0.33	0.09±0.14	0.07±0.12	0.13±0.28	0.02±0.06	0.004±0.015	0.06±0.18	0.03±0.06
	Swab	0.02±0.06	0.38±0.71	0.67±0.97	1.34±1.23	0.82±0.65	0.61±0.59	0.90±0.73	0.91±0.66
	Stats				ac	ac		ac	ac

Values presented as mean ± standard deviation. n = 13-15; a, $p < 0.05$ for milk vs. swab; b, $p < 0.05$ for milk vs. day 1; c, $p < 0.05$ for swab vs. day 1.

Table S2 (cont.). Percentage abundance of microbial families present in mare's milk and foal rectal swabs.

Families		Day 1	Day 3	Day 5	Day 7	Day 9	Day 11	Day 13	Day 15
Proteobacteria (cont.)									
Enterobacteriaceae	Milk	69.30±13.47	46.96±23.18	68.40±15.17	60.55±20.42	56.41±18.73	65.89±16.00	60.87±20.25	62.06±18.53
	Swab	62.01±31.45	50.88±28.07	22.03±16.23	4.06±4.98	1.02±0.53	1.11±1.18	1.05±0.84	0.82±0.53
	Stats			ac	ac	ac	ac	ac	ac
Moraxellaceae	Milk	1.17±1.54	1.16±1.67	0.55±0.94	1.22±2.02	1.37±1.06	0.93±1.24	4.35±12.22	2.05±2.26
	Swab	1.63±4.20	0.05±0.19	0.02±0.10	0.03±011	0.03±0.09	0.39±1.32	0.01±0.03	0.04±0.08
	Stats								
Pasteurellaceae	Milk	0.80±1.11	2.08±3.80	0.53±1.05	1.29±1.93	1.31±2.08	0.83±0.94	1.39±2.00	1.69±2.74
	Swab	12.90±32.58	0.36±0.57	0.30±0.59	0.38±1.06	0.43±0.65	0.62±1.38	0.51±0.90	0.17±0.25
	Stats		c	c	c	c	c	c	c
Sutterellaceae	Milk	0.58±031	0.26±0.34	0.23±0.36	0.28±0.41	0.53±0.38	0.28±0.43	0.44±0.40	0.44±0.33
	Swab	0.15±0.20	0.50±1.79	1.66±4.71	1.81±2.18	1.17±1.15	0.87±0.96	1.24±2.08	1.22±1.38
	Stats	a							
Phylum Actinobacteriota									
Corynebacteriaceae	Milk	0.89±1.23	1.32±1.61	0.59±1.17	1.10±1.44	1.19±1.21	0.49±0.55	1.11±2.15	1.90±3.98
	Swab	0.62±1.54	0.01±0.02	0.06±0.17	0±0	0±0	0.21±0.45	0.56±1.29	0.70±0.87
	Stats								
Eggerthellaceae	Milk	0±0	0.01±0.03	0.01±0.03	0.02±0.05	0.01±0.02	0.03±0.07	0±0.02	0.03±0.09
	Swab	0.01±0.03	0.11±0.21	0.18±0.23	0.18±0.24	0.12±0.13	0.14±0.20	0.29±0.17	0.32±0.26
	Stats							ac	ac
Intrasporangiaceae	Milk	1.29±2.61	3.48±4.80	1.03±1.49	1.33±2.59	1.68±2.62	1.49±2.96	1.13±1.97	1.21±1.37
	Swab	0.05±0.15	0±0	0±0	0.03±0.12	0±0.01	0±0	0±0	0±0
	Stats		a						
Micrococcaceae	Milk	0.47±1.30	2.36±2.08	0.78±3.00	0.05±0.12	1.97±2.20	1.52±1.77	2.25±2.30	1.30±1.08
	Swab	0.29±0.71	0.24±0.34	0.35±0.36	0.14±0.21	0.05±0.13	0.03±0.10	0±0	0.03±0.10
	Stats		ab		a	a		ab	
Phylum Fibrobacterota									
Fibrobacteraceae	Milk	0.55±0.33	0.23±0.28	0.45±0.33	0.31±0.37	0.35±0.38	0.43±0.39	0.34±0.31	0.41±0.27
	Swab	0.19±0.18	0.07±0.14	0.01±0.02	0.02±0.05	0.13±0.43	0.34±0.30	0.14±0.18	0.15±0.24
	Stats			a					
Phylum Fusobacteriota									
Fusobacteriaceae	Milk	1.82±0.72	1.54±1.61	1.73±0.77	1.44±0.96	1.67±0.82	1.44±0.70	1.40±0.59	1.44±0.86
	Swab	0.75±0.79	7.69±10.44	9.55±15.50	10.37±17.15	10.57±13.97	11.25±14.67	7.22±10.74	3.04±3.80
	Stats	a							

Values presented as mean ± standard deviation. n = 13-15; a, $p < 0.05$ for milk vs. swab; b, $p < 0.05$ for milk vs. day 1; c, $p < 0.05$ for swab vs. day 1.

Table S2 (cont.). Percentage abundance of microbial families present in mare's milk and foal rectal swabs.

Families		Day 1	Day 3	Day 5	Day 7	Day 9	Day 11	Day 13	Day 15
Phylum Euryarchaeota									
Methanobacteriaceae	Milk	0.25±0.26	0.14±0.13	0.25±0.21	0.16±0.21	0.20±0.25	0.18±0.18	0.14±0.16	0.22±0.26
	Swab	0.13±0.23	0.01±0.06	0.01±0.02	0.01±0.02	0.02±0.04	0.04±0.09	0.10±0.13	0.06±0.09
	Stats			a					
Phylum Spirochaetota									
Spirochaetaceae	Milk	0.53±0.57	0.24±0.38	0.57±0.56	0.27±0.26	0.34±0.36	0.32±0.38	0.34±0.39	0.51±0.63
	Swab	0.13±0.16	0.02±0.04	0.03±0.03	0±0.01	0.04±0.10	0.38±0.53	0.37±0.36	0.44±0.74
	Stats			a					

Values presented as mean ± standard deviation. n = 13-15; a, $p < 0.05$ for milk vs. swab; b, $p < 0.05$ for milk vs. day 1; c, $p < 0.05$ for swab vs. day 1.

Table S3. Study Sample Numbers

Sample Type	Day 1	Day 3	Day 5	Day 7	Day 9	Day 11	Day 13	Day 15
**Milk**	14	15	15	14	15	15	14	15
**Rectal Swab**	15	15	15	15	15	15	14	13

Samples Not Collected: Day 1: Horse 113 Milk; Day 7: Horse 115 Milk; Day 13: Horse 111 Swab, Horse 113 Milk; Day 15: Horse 102 Swab, Horse 105 Swab.

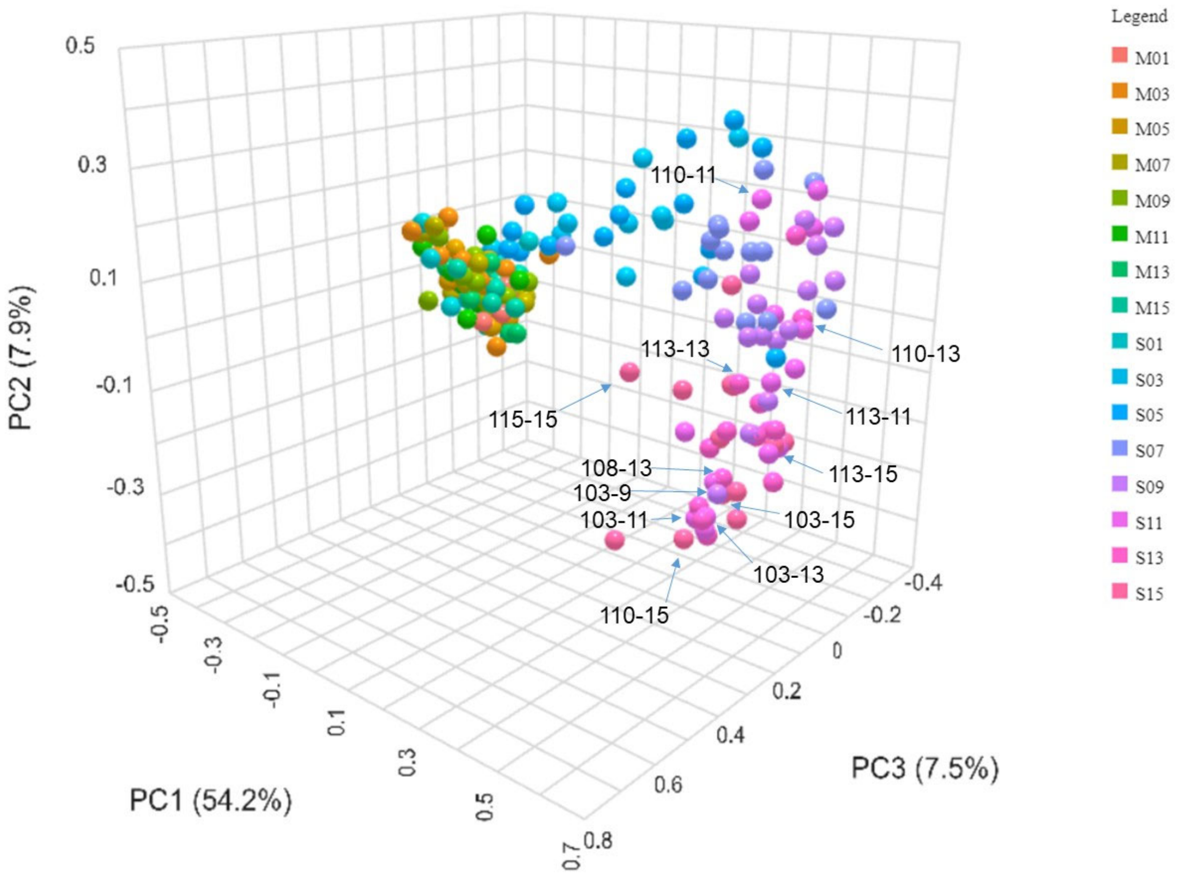

Figure S1. Distribution of diarrhea and antibiotic-treated samples within the principal coordination analysis. A subset of rectal swab samples were collected from horses with diarrhea (Horses 108, 110, 113, and 115); one horse received antibiotics for an umbilical infection (Horse 103). Swabs noted represent days of diarrhea or days of antibiotic treatment. Color categories for days and sample type are noted (M, mare's milk; S, foal rectal swab; days post-foaling are accompanying numbers). Naming convention is "Horse number – Day."

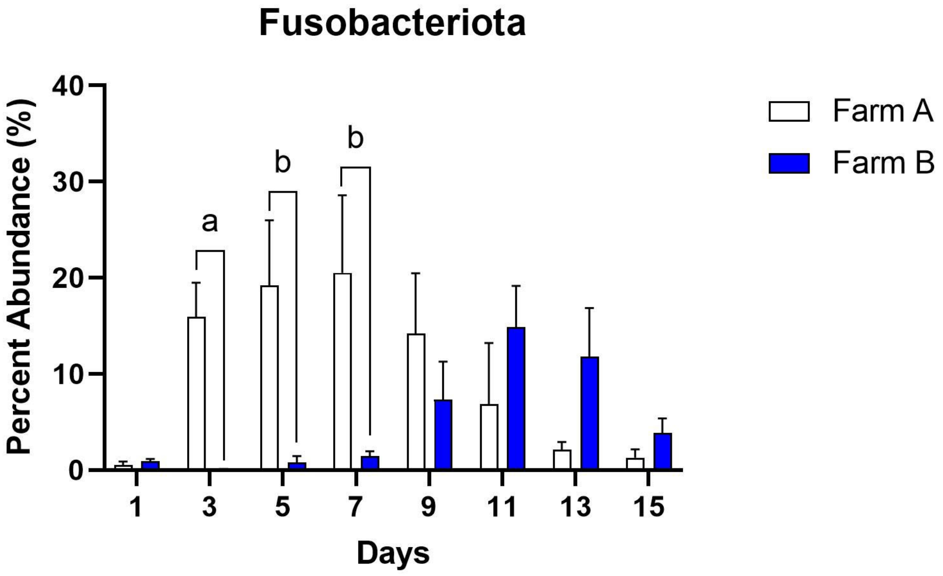

Figure S2. Farm-specific Differences in Phylum *Fusobateriota* Abundance for Foal Rectal Swabs. There was only one farm-specific difference in microbial populations for all samples collected - *Fusobacteriota*. n = 5-8 by day x Farm; ANOVA (Sample Type x Day x Farm) with post-hoc Tukey's comparisons as $p_{adjusted}$ differences by type-day-farm; a, $p < 0.05$; b, $p < 0.01$.
